# Elongation of Cervix Uteri

**Published:** 1847-04

**Authors:** 


10. *Elongation of the Cervix Uteri.*—Prof. Gilman, amongst other interesting obstetrical cases, reported in the Annalist, gives the following:

*Case* 1.—I was requested by Dr. Barker to see a puerperal patient with him. Found Mrs. B., on the sixth day after her confinement, much exhausted and suffering from various nervous symptoms. She had effusion of serum into the peritoneum, and the legs were considerably enlarged by œdema: on examination per vaginum, the os uteri was found just within the labia. On passing the finger up into the posterior "*cul de sac*," it appeared to be from four to five inches long; and in feeling forward, the cervix was made out, elongated and cylindrical, about an inch and a half in diamater, rather larger above, though the difference was not great. The os was circular—the size of a shilling; the division into anterior and posterior lip was less distinct than I ever remembered to have found it in a recently delivered woman.

Dr. Barker informed me that the neck had once or twice protruded from the vulva, on the patient making some effort, straining or the like. Under treatment directed to her other symptoms, the patient recovered.

*Case* 2.—January 17. Saw in consultation with Dr. P. O'-Reilly, Mrs. S., aged 27, married three years—never pregnant. She had suffered for some time with pain and tenderness in the hypograstic region, which on examination, was believed to depend on irritation of the bladder. The particulars of this diagnosis, not being to the present purpose, I will not detail: suffice it to say, that on making a vaginal examination, the cervix uteri was found to project into the vagina about two inches. It was small and quite pointed, the os round and of a size scarcely to admit a common probe. Contrary to what might have been expected, this female, though she menstuated sparingly, suffered but little at her turns—certainly not to a degree that would make hers a case of dysmenorrhœa.

*Remarks.*—Elongation of the cervix uteri is rare—that of one lip is exceedingly so. In the hasty and imperfect reference to authorities which I have been able to make, I find no distinct notice of the disease as effecting one lip, except in an extract from Leroux, inserted in "Boivin and Dugès on the Uterus."

He speaks of it as occurring to some women during pregnancy, but disappearing on the approach of labor. Elongation of the whole cervix is more common. Segard reports a case, where the elongated cervix was mistaken for a polypus, a ligature applied, a fatal peritonitis and death were the results. Bichat (Anat, Descrip., Vol. V., p. 282) speaks of having found the cervix elongated in several bodies examined post mortem.—He also details a case from Buisson, where the elongated cervix was mistaken for prolapsus, and a pessary used! A degree of the elongation noticed in the case of Mrs. S. is familiar to obstetricians, and, together with the pointed shape and the small size of the os, is noticed by Churchill and others as a frequent concomitant of dysmenorrhœa.

The opinion of Mackintosh, that a stricture or obliteration of the os was the frequent cause of dismenorrhœa, and that removing the stricture would often cure the disease, has never made a strong impression on the minds of the profession. That the theory is well founded, and the practice in very many cases valuable, I have long believed; and I now repeat the opinion, elsewhere expressed, that in every case of dysmenorrhœa of great severity occurring in a married woman, especially if accompanied—as it so frequently is—by barrenness, a careful vaginal examination should be made, and if the os uteri be found small, (too small, for example, to admit a moderate sized female catheter,) careful attempts made to dilate it. This practice will, I know from an experience of now many years, be followed by a cure of the dysmenorrhœa, and, what is often to the patient more acceptable, a removal of the dreaded reproach of sterility.

The closing paragraph is worthy the particular attention of the profession. But what immorality would there be in this practice in cases of unmarried women, as intimated in a note?

